# Age and sex-adjusted incidence and yearly prevalence of multiple sclerosis (MS) in Mazandaran province, Iran: An 11-years study

**DOI:** 10.1371/journal.pone.0235562

**Published:** 2020-07-02

**Authors:** Hamed Cheraghmakani, Seyed Mohammad Baghbanian, Reza HabibiSaravi, Arash Azar, Fariba Ghasemihamedani

**Affiliations:** 1 Neurology Department, Boualicina Hospital, Mazandaran University of Medical Sciences, Sari, Iran; 2 Multiple Sclerosis Clinic, Boualicina Hospital, Mazandaran University of Medical Sciences, Sari, Iran; 3 School of Public Health, Faculty of Medicine, University of Sydney, Sydney, New South Wales, Australia; 4 School of Health Management and Information Sciences, Iran University of Medical Sciences, Tehran, Iran; 5 Mazandaran Multiple Sclerosis Society, Sari, Iran; University of Oxford, UNITED KINGDOM

## Abstract

**Introduction:**

The incidence rate of MS is a valuable indicator of the recent changes in the risk of this disease, and it is widely implicated for health planning purposes.

**Objectives:**

This study aims to determine the MS incidence over the past eleven years in Mazandaran province and to compare it with the other parts of Iran and the world.

**Materials and methods:**

This retrospective study is conducted in Mazandaran province by using registered data in the files of the patients with their consent. The yearly crude incidence rates, age, and sex-specific incidence rates and directly standardized incidence rates of this population are calculated, and the temporal changes in the incidence rates are analyzed.

**Results:**

662 (26%) male patients with the mean (SD) age of 32.6 (9.48) and 1884 (74%) female patients with the mean (SD) age of 31.9 (9.15) are studied. The direct standardized incidence rate of MS was 3.28 in 100.000 in 2008 and reached 4.17 in 100.000 in 2018, and this increase was significant (p<0.05). Also, the yearly prevalence of MS increased from 24.4 to 72.5 in this period.

**Conclusions:**

The MS incidence has increased in Mazandaran. The potential role of some genetic or environmental factors needs further investigation.

## Introduction

The epidemiologic pattern of multiple sclerosis constantly changes around the world [1]. In the early stages of the MS epidemiologic studies, Kurtzke categorized countries into three different categories of MS prevalence. Based on this categorization, low MS prevalence countries are those with less than five cases per 100,000 population, medium prevalence countries have 5 to 30 MS cases per 100,000, and high prevalence countries have over thirty MS patients per 100,000 population. According to the Kurtzke categorization method, Iran was among the low prevalence countries [2].

Many studies were conducted to test this hypothesis. A study in Mazandaran province in 2007 showed that the point prevalence of MS in this region is 20.1 per 100,000 [3]. Other concurrent studies in Isfahan [4, 5] and other regions in Iran [6] confirmed the findings of this study and led to a change in MS prevalence Map of Iran, and moving this country from a low prevalence country to a medium prevalence country [7].

As MS is not a disease of the targeted health surveillance system, the world MS epidemiologic data is gathered from separate observational studies [8]. Different studies throughout the world [8, 9], as well as studies conducted in Iran [6, 10], demonstrate an increasing temporal trend in the incidence of this disease. Mazandaran is located in central North of Iran, and South of the Caspian Sea, between the latitudes 36.477504 and 36.892709. Mazandaran is a popular destination for immigrants because of its temperate climate and plenty of job opportunities. Therefore, Mazandaran has a heterogeneous population and the third-highest population density after Tehran and Gilan provinces in Iran [11]. We can see a four-times increase in the prevalence of MS in Mazandaran in a period of eleven years from the initial study in this province in 2008 [3] to the recent study in 2018 [12].

Since the incidence rate is a valuable indicator of recent changes in the risk of MS, this study aims to determine the incidence of this disease over the past eleven years in Mazandaran province and to produce age-standardized incidence rates that are comparable with other parts of Iran and the world using the direct standardization method. In addition, we intend to find the annual prevalence, incidence, and specific disease incidence in sex and age groups for operational use in the health care system. Finally, as the temporal changes in the incidence rates can be caused by changes in the demographic characteristics of the region over this period, our last objective is to fit a regression model to analyze the temporal changes in the incidence rates by adjusting for age and sex variables.

## Materials and methods

This retrospective study is conducted in 2019 in Mazandaran province. According to the latest census in 2016 and the population prediction of 2018, the total population of Mazandaran was 3,332,556 people in 2018 [11]. The period of this study was March 21, 2008, to March 20, 2019 (March 21 is the beginning of the Iranian new year according to the Iranian calendar).

The Mazandaran Province MS Society (MPMSS) provided the data of all the MS patients in this province. This non-governmental organization collaborates with Mazandaran University of Medical Sciences, academic societies such as the Mazandaran Neurology Association, and other governmental healthcare organizations closely to plan and manage issues related to MS patients. Mazandaran MS patients can only access to governmentally subsidized MS medications through registration in the Mazandaran MS Society and obtaining a referral to the Mazandaran University of Medical Sciences for medical and pharmaceutical services. Hence, MPMSS is the only registry of definite diagnosed MS patients (based on the McDonald’s diagnostic criteria) [1, 13] in the Mazandaran province [14].

The registration process in MPMSS includes the submission of patients’ certificate of MS diagnosis as well as their national identity cards and birth certificates. Also, patients need to fill a questionnaire including questions about their personal, disease-related, and demographic information (name, date of birth, gender, immigration status and date, family history of MS, and marital status).

Patients need to complete questionnaires under the supervision of a qualified general practitioner (for confirmation and registration). In the case of patients with disabilities or minors (under 18 years old), their next of kin/guardians need to complete the questionnaires. Also, patients can voluntarily sign a consent form to let MPMSS use their information in future research projects anonymously. All registered patients agreed to the use of their data for research purposes. Patient Information Sheets and Consent Forms (PISCFs) contain detailed explanations of the possible purposes for the use of this information, and their agreement or disagreement to sign this form will not affect their access to their medical and pharmaceutical services. After submission, patients receive their MPMSS identification card and a referral to the Mazandaran University of Medical Sciences for receiving their MS medical and pharmaceutical benefits. Patients or their carers/guardians need to keep their contact information and addresses updated.

We obtained the approval of the Human Research Ethics Committee (HREC) for this study as follows: HREC number: IR.MAZUMS.REC.1398.4778–4/20/2019.

The ascertainment degree of the data was 99% as our inquiry from the Iranian MS society showed that while there are no patients from other provinces who are registered in the Mazandaran MS society, 99% of MS patients who live in the Mazandaran province are registered at this registry, and the remaining registered in neighboring provinces. We extracted the required information from all the patients’ files. All the patients had valid consent forms, and there were no dropouts. Even the patients who had passed away or immigrated out of the region were included. In the case of incomplete registered data, we arranged a face to face or telephone interview with the patients or their carers /guardians. A team of data administrators reviewed and confirmed the validity and correctness of the entrees and fixed the mistakes.

We used the date of the diagnosis of the disease by a neurologist for calculation of the incidence rates to decrease the measurement bias. We defined the incidence rate as the number of new cases in 100,000 average population of the region in a year. The trend of the changes in the incidence rates was analyzed by using the Poisson regression method.

To calculate the results, we obtained Mazandaran population statistics from the latest census in this province from 2006 to 2017 and the population prediction of 2018, published in the Iran Population Statistics portal [15] and the Mazandaran Province Birth Registry [16]. This information was used to calculate the yearly prevalence and the incidence of MS in this province.

We performed the analysis of the dataset by Stata version 15. We calculated the age-standardized incidence rates and their variances, standard errors, and the 95% confidence intervals using the direct standardization method and the WHO world standard population [17] to provide comparable rates. We compared the directly standardized incidence rates in 2008 and 2018 by computing a chi-squared statistic from the difference between the rates and the variance of this difference. Cases were grouped by sex and age, and Poisson regression models were fitted to the data to derive estimates of incidence rate trends in the age groups from 2008 to 2018. In our first model, we fitted a Poisson regression model with incidence rates as the outcome variable and year as the exposure variable, and in the second fitted model, we added sex and age group covariates to our model and compared the level of fitness in them.

## Results

The total number of registered patients was 2546 people comprised 662 (26%) male patients with the mean (standard deviation(SD)) age of 32.6 (9.48) and 1884 (74%) female patients the mean (SD) age of 31.9 (9.15). Table 1 shows this yearly increase from 2008 to 2018 in different age and gender groups.

**Table 1:** Sex-specific annual incidence and prevalence per 100,000 population and sex risk ratio of MS in Mazandaran, Iran.

Year	Population			Cases				Incidence				Prevalence
	Male	Female	Total	Male	Female	Total	F/M ratio	Male	Female	Crude Incidence Rate	Directly Standardized Rate	
2008*	1,501,155	1,489,944	2,991,099	32	83	115	2.6	2.1	5.6	3.8	3.3	24.4
2009	1,512,206	1,500,912	3,013,118	36	106	142	2.9	2.4	7.1	4.7	3.9	29.0
2010	1,524,392	1,513,007	3,037,398	28	119	147	4.3	1.8	7.9	4.8	4.2	33.6
2011	1,542,730	1,531,208	3,073,938	36	124	160	3.4	2.3	8.1	5.2	4.3	38.4
2012	1,557,568	1,547,123	3,104,690	56	160	216	2.9	3.6	10.3	7.0	6.0	44.9
2013	1,568,476	1,557,797	3,126,273	50	128	178	2.6	3.2	8.2	5.7	4.8	50.3
2014	1,581,502	1,571,585	3,153,087	47	146	193	3.1	3.0	9.3	6.1	5.1	56.0
2015	1,595,004	1,585,034	3,180,038	44	146	190	3.3	2.8	9.2	6.0	5.0	61.5
2016	1,653,998	1,629,584	3,283,582	52	141	193	2.7	3.1	8.7	5.9	5.2	65.5
2017	1,685,424	1,660,546	3,345,970	51	154	205	3.0	3.0	9.3	6.1	5.4	71.2
2018	1,717,447	1,692,096	3,409,543	53	114	167	2.2	3.1	6.7	4.9	4.2	72.5

**Fig 1:**
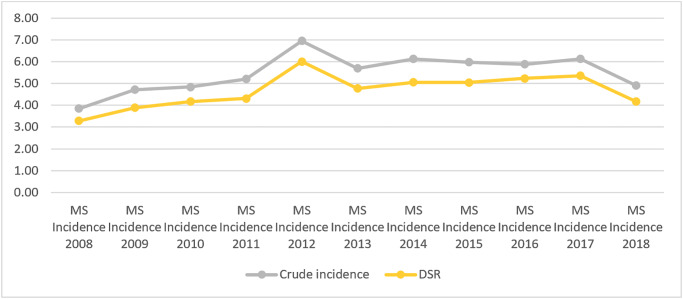
Crude and directly standardized incidence rates from 2008 to 2018.

Also, Fig 1 illustrates this trend from 2008 to 2018 in crude and directly standardized incidence rates. As can be seen, in 2012, we had the highest incidence rate for MS. Also, we can see that in 2018, we have a decrease in the incidence of this disease despite the increasing trend of the previous years.

There are many variations in age groups’ incidence rates in different years. Table 2 illustrate details of the incidence in age groups by year.

**Table 2:** Number of MS new cases and age-specific incidence by year.

**a** Number of new cases												
**Age groups Year**	**10–14**	**15–19**	**20–24**	**25–29**	**30–34**	**35–39**	**40–44**	**45–49**	**50–54**	**55–59**	**60–65**	**>65**
2008*	0	7	22	20	19	21	10	5	7	4	0	0
2009	0	14	30	41	22	11	11	13	0	0	0	0
2010	1	15	23	26	25	22	16	11	7	1	0	0
2011	0	10	37	35	27	22	22	5	2	0	0	0
2012	2	23	36	36	37	44	24	9	4	1	0	0
2013	0	10	34	34	36	24	25	12	3	0	0	0
2014	0	12	40	49	38	25	13	11	3	1	1	0
2015	2	8	28	38	54	28	16	7	5	1	2	1
2016	0	14	32	35	43	32	20	5	5	4	3	0
2017	1	13	21	51	35	40	20	10	7	5	1	1
2018	3	3	23	37	40	40	6	10	4	1	0	0
***b*** Age-specific incidence												
2008*	0.00	3.02	6.85	5.82	7.06	8.99	4.50	2.57	4.19	3.19	0.00	0.00
2009	0.00	5.99	9.28	11.84	8.11	4.67	4.92	6.63	0.00	0.00	0.00	0.00
2010	0.17	6.37	7.05	7.45	9.15	9.27	7.10	5.56	4.12	0.78	0.00	0.00
2011	0.00	4.19	11.21	9.91	9.76	09.16	9,64	2.50	1.16	0.00	0.00	0.00
2012	0.33	9.55	10.80	10.09	13.24	18.14	10.41	4.48	2.30	0.77	0.00	0.00
2013	0.00	4.12	10.13	9.46	12.79	9.83	10.77	5.92	1.72	0.00	0.00	0.00
2014	0.00	4.90	11.81	13.51	13.39	10.15	5.55	5.41	1.70	0.75	1.02	0.00
2015	0.32	3.24	8.20	10.39	18.86	11.27	6.77	3.41	2.81	0.75	2.01	0.47
2016	0.00	6.79	13.61	10.84	11.79	11.00	8.11	2.15	2.49	2.37	2.37	0.00
2017	0.15	6.18	8.77	15.50	9.42	13.50	7.96	4.22	3.42	2.91	0.78	0.40
2018	0.45	1.40	9.42	11.03	10.56	13.25	2.34	4.14	1.92	0.57	0.00	0.00

To compare the fitted models for the temporal trend in incidence rates, the goodness of fit measures such as deviance, Pearson chi-square, Akaike information criterion (AIC), Bayesian information criterion (BIC), and the pseudo R2 statistics show that the preferred model is the model with year, sex, and age variables. The Pseudo R2 statistic also shows that this model explains about 62.3% of the data variability while, in comparison, the model without age and sex variables only explains 1.18% of the variability of the data. Therefore, adding sex and age variables to the model improves the model.

Our model shows very strong evidence (p<0.001) for associations between MS incidence and age categories 15–19, 20–24, 25–29, 30–34, 35–39, 40–44, 45–49, and 50–55 when the results are adjusted for sex and year variables. There is also evidence for this association in the age group 55–59 (P<0.05). We cannot see an association between these variables for the age group 60–64, and there is evidence that the MS incidence rate in the age group +65 declines compared to the baseline age group.

Table 3 summarizes the ratio of the MS incidence rates in different age groups to the MS incidence rate of our baseline age group (10–14 years) when adjusted for sex and age variables. (Table 3).

**Table 3:** The ratio of MS incidence in different age groups to the baseline age group (10–14 years old) when adjusted for sex and year variables.

Age group	Incidence rate to base age group	95% Confidence Interval
15–19	12.3	6.6, 25.5
20–24	24.0	12.4, 46.6
25–29	25.1	13.0, 48.6
30–34	28.5	14.7, 55.2
35–39	27.8	14.3, 54.0
40–44	17.9	9.2, 35.0
45–49	10.7	5.4, 21.2
50–54	5.9	2.9, 12.0
55–59	2.9	1.3, 65.0
60–64	1.5	0.6, 4.0
Over 65	0.2	0.0, 1.0

The incidence of MS is higher in most groups compared to the reference group (which is the youngest group). However, the incidence is clearly highest in the 30–34 age group and is declining after that, indicating that there is not a simple linear relationship between the incidence rate and age when adjusted for sex and year variables up to 65 years of age. We can also see that the rates of the increase in the incidence of this disease increase up to the age group 30–34, and then declines. At the age group 60–64, the incidence of MS is 1.5 times higher than the baseline age group of 10–15, and this incidence rate declines further for the age group over 65 to 0.2 of the incidence in our base age group. Our fitted model also shows that the MS incidence in females is 3.1 times higher than males CI (2.8, 3.5) when controlled for variables age and year. This is a statistically significant increase (p<0.001).

The results suggest a significant association between the variable year, and *age and sex-specific MS incidence rates* (p<0.001) when controlled for age and sex variables.

**Fig 2:**
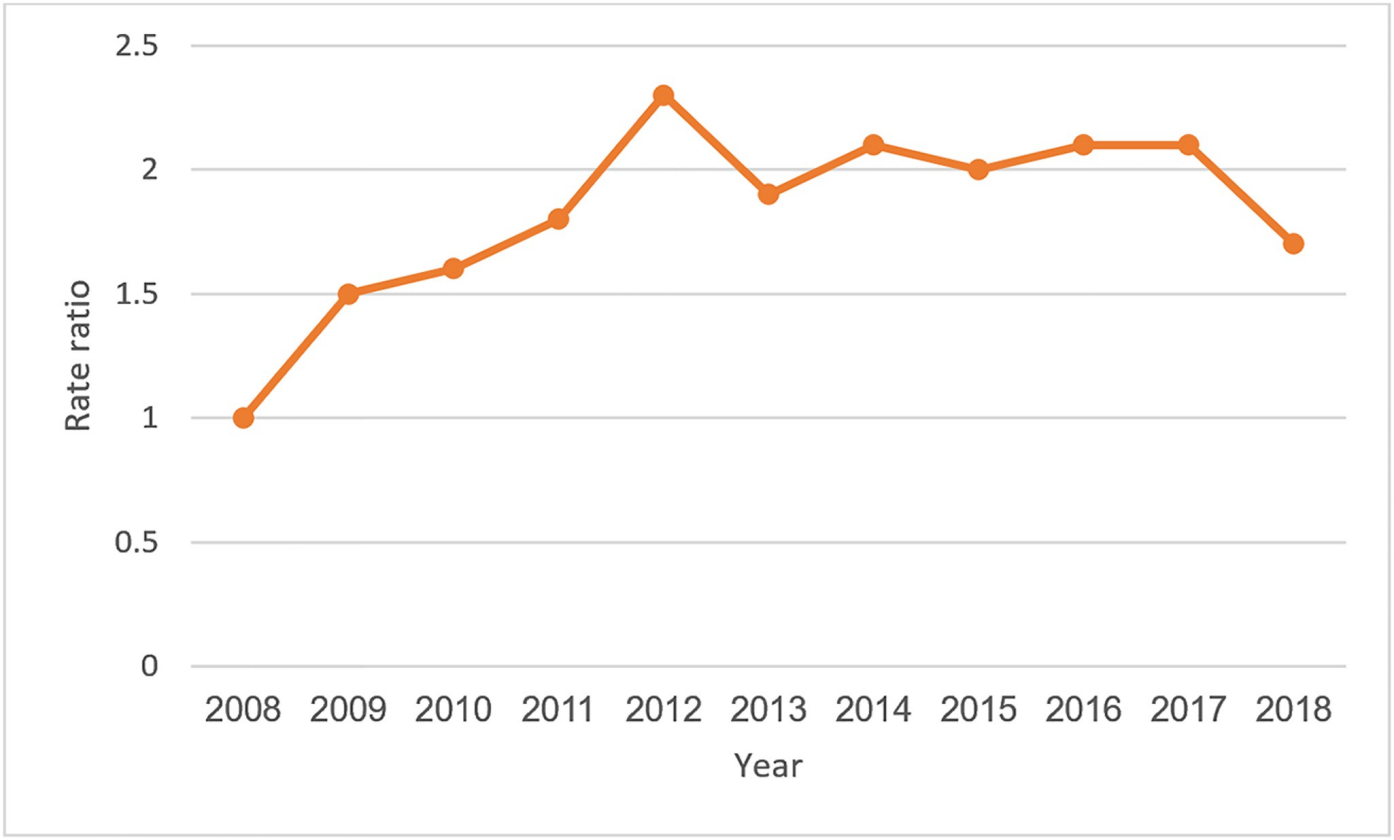
Age and sex adjusted incidence rate ratios compared to the baseline year (2008).

The incidence rate of MS Compared to 2008, show very strong evidence of an increase in all subsequent years (Table 4). This increase is plotted in Fig 2.

**Table 4:** The ratio of MS incidence to the baseline year (2008) when adjusted for sex and age variables.

Age group	Incidence rate to base age group	95% Confidence Interval
2009	1.5	1.1, 1.9
2010	1.6	1.3, 2.1
2011	1.8	1.4, 2.2
2012	2.3	1.9, 3
2013	1.9	1.5, 2.4
2014	2.1	1.6, 2.6
2015	2.0	1.6, 2.5
2016	2.1	1.7, 2.6
2017	2.1	1.7, 27
2018	1.7	1.3, 2.2

Based on the Poisson regression model, if we consider year as a continuous variable, the age and sex specific MS incidence has a yearly increase of 0.04, CI (0.03, 0.06), and P-value<0.001).

Finally, the chi-square test shows that there is evidence that the directly standardized incidence rate in 2018 is higher than this rate in 2008 with (P-value<0.05).

**Fig 3:**
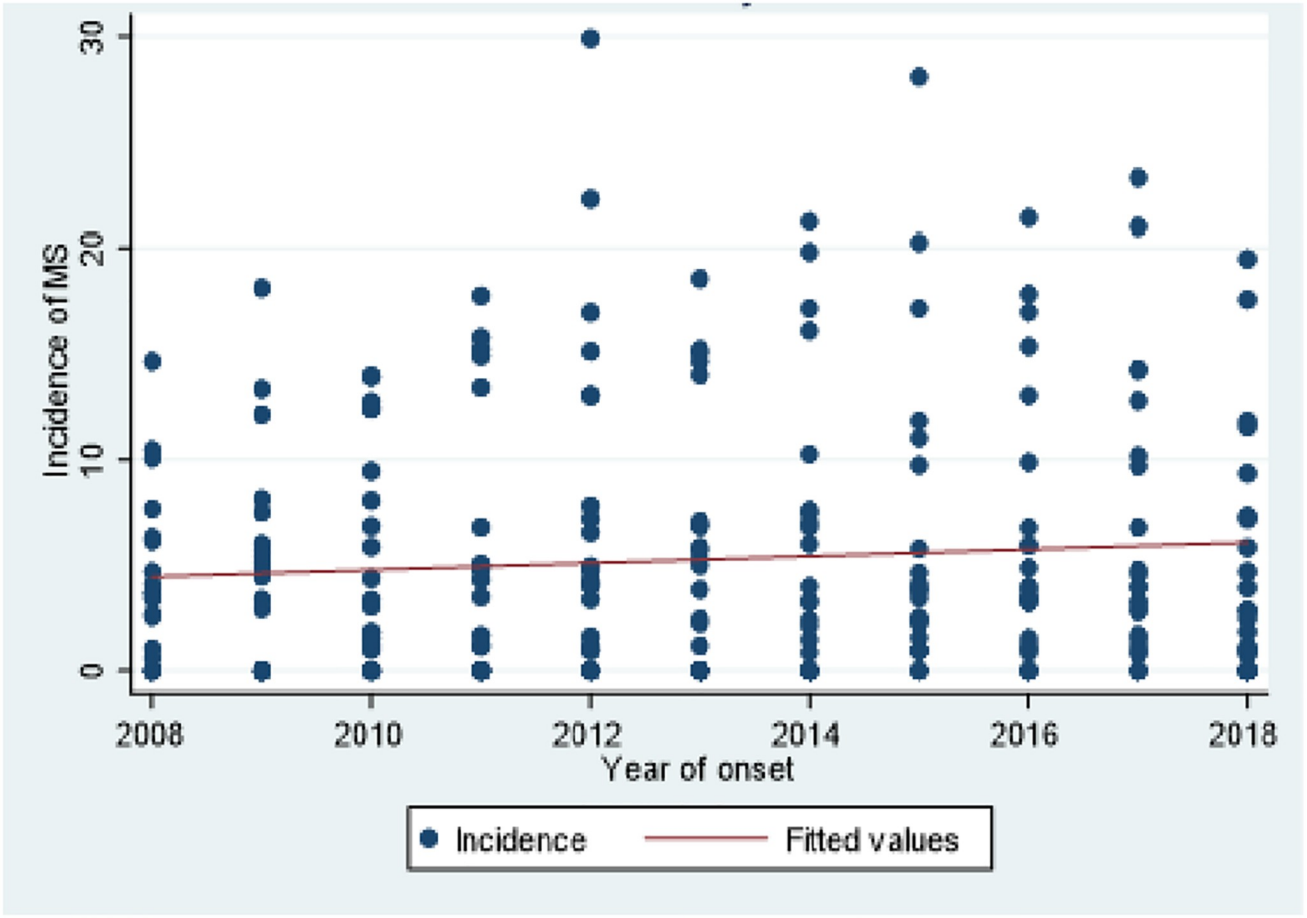
Sex and age-specific MS incidence vs. year of onset.

As can be seen in Fig 3, there is an association between the year of the onset of the disease and the age and sex adjusted incidence rate of this disease with a slight upward slope.

## Discussion

MS cases increased in Mazandaran province over the past 11 years, with the highest prevalence age groups of 20 to 40 years old. The annual incidence of the disease has increased over the last 11 years with a mild variation in different years, and there has been a 3.6-fold increase in the cumulative prevalence of MS from 20.1 [3] in 2007 to 72.5 per 100,000 in 2018 [12]. There was an increase in female to male ratio of MS incidence during the study period. The increase in the incidence rate for females was more than males with roughly two folds in females compared to 1.5 times increase in males.

There are many reasons for this variation in incidence rates during the recent years of the study period. One possible explanation can be attributed to the patients’ tendency to go to neighboring provinces to access healthcare services because of proximity to their home location, especially in the west of Mazandaran province. Another reason may be the proximity of Mazandaran province to Tehran, the capital of Iran, with a significantly more high-quality health care facilities. Thus, many MS patients prefer to follow their treatment there. The inquiry from the Iranian MS society on the number of registered MS patients who reside in Mazandaran showed that 99 percent of Mazandaran MS patients are registered at the Mazandaran MS Society and included in this study and the remaining registered in neighboring provinces. While there are no patients from other provinces, who are registered at Mazandaran MS society. This fact may slightly underestimate the incidence rate of MS in Mazandaran. Finally, the changes in diagnostic criteria and methods that lead to higher diagnostic sensitivity for definite cases of multiple sclerosis can be considered as one of the reasons for this variation Mazandaran MS Society uses Macdonald criteria as its diagnostic tool. This tool has had several revisions in past years. Specifically, MRI was added as a criterion to the most recent version of Macdonald criteria, so the sensitivity of the diagnosis of MS has increased. This may result in an increase in the incidence rate of MS after this new version was adapted by Mazandaran MS society in 2010 [1].

The increasing trend of MS incidence in Mazandaran province is almost similar to the other parts of Iran, such as Isfahan [10], Shiraz [18], Tehran [19], and Southern parts of Iran [20]. An increase in the MS incidence has been reported from other countries like the United Kingdom [21], Japan [22], Wales [23], and France [24]. Although a decrease in the incidence rate of MS has been reported in countries such as Sweden in previous studies [25], subsequent studies showed a high nationwide incidence of multiple sclerosis in that country [26]. The increasing trend of MS incidence in the world may be partly attributed to the improvement of diagnostic criteria for MS, promotion of the general population’s knowledge, physicians’ alertness, and easier access to high-quality health services. However, due to different study methodologies and diagnostic criteria, a comparison of the results with these countries should be made with caution.

Although in the initial study of Kurtzke [27], Iran with latitudes lower than 42° is considered a low-risk area for MS. Subsequent studies resulted in the correction of the place of Iran and placed it in the medium-risk prevalence area [28]. However, according to the results of recent studies, Iran may need to be placed in the high-risk prevalence area for MS [12, 19].

The increasing trend of MS in Iran may be attributable to genetic factors, as suggested by a study in India [29]. Evidence showed the MS disease is more prevalent in Parsi Indians (Persian migrants) than in the general population [30]. A study in England showed that the rate of MS was higher in Parsis (Persian migrants in India) than ethnic Indians [31]. Another study in Norway also showed MS prevalence in immigrants from the Middle East (mainly from Iran) was higher than its rates in the other non-Western immigrants. This study indicated that Iranian patients were similar to the Norwegian population in terms of genetic susceptibility [32]. Many studies in Iran demonstrated predisposing genetic factors and gene presentation and polymorphism in peripheral blood cells [33–35].

These findings emphasize that the MS is resulting from interactions between both environmental and genetic factors [36] Therefore, we can suppose that Iranian people have special genetic predisposing factors, which are triggered under particular environmental conditions. Different environmental factors such as geo-climate changes, nutrition, psycho-behavioral stressors such as recent international sanctions [37], infections, and exposure to hazardous materials like organic solvents are potentially involved in the pathogenesis of MS [38].

## Conclusion

Mazandaran is placed in a high-risk area for MS, and the incidence of MS in this province has been increasing both in females and males over the past 11 years. The increasing trend of MS in Mazandaran is similar to this trend in other parts of Iran and the world. Our findings support that this is mostly a real increase. However, this increase might be due to some genetic or environmental factors. Therefore, further studies need to be conducted to identify those factors that affect the MS incidence, especially the unique genetic pattern in Iran. We believe that genetic factors alone cannot cause this considerable increase in the incidence of MS because we do not seem to have a significant genetic change in this period. Urbanization, environmental stressors, exposure to hazardous materials, and nutrition can potentially be some of the more effective factors.
